# Clinical and genetic features of UNC13D deficiency with hypogammaglobulinemia

**DOI:** 10.3389/fimmu.2025.1628507

**Published:** 2025-08-18

**Authors:** Linyan Xiong, Qin Zhao, Qian Zhao, Zhiyong Zhang, Yunfei An, Xuemei Tang, Hirokazu Kanegane, Xi Yang, Xiaodong Zhao

**Affiliations:** ^1^ Department of Rheumatology and Immunology, Children’s Hospital of Chongqing Medical University, Chongqing, China; ^2^ National Clinical Research Center for Child Health and Disorders, Ministry of Education Key Laboratory of Child Development and Disorders, Children’s Hospital of Chongqing Medical University, Chongqing, China; ^3^ China International Science and Technology Cooperation Base of Child Development and Critical Disorders, Children’s Hospital of Chongqing Medical University, Chongqing, China; ^4^ Chongqing Key Laboratory of Child Rare Diseases in Infection and Immunity, Children’s Hospital of Chongqing Medical University, Chongqing, China; ^5^ Department of Child Health and Development, Graduate School of Medical and Dental Science, Tokyo Medical and Dental University (TMDU), Tokyo, Japan

**Keywords:** hemophagocytic lymphohistiocytosis, familial hemophagocytic lymphohistiocytosis, UNC13D, hypogammaglobulinemia, antibody deficiency

## Abstract

**Background:**

UNC13D deficiency is the most common form of familial hemophagocytic lymphohistiocytosis (FHL) in Asia. Hypogammaglobulinemia is a rare phenotype observed in both patients with FHL3 and sporadic hemophagocytic lymphohistiocytosis (HLH). Our observations suggest that UNC13D deficiency with hypogammaglobulinemia presents a distinct clinical phenotype compared to other HLH patients. This finding provides valuable clinical insights and may contribute to a more comprehensive understanding of the disease, highlighting the need for further investigation into its genetic and clinical characteristics.

**Methods:**

We retrospectively analyzed the clinical features of five patients with UNC13D deficiency with hypogammaglobulinemia at our center, along with a literature review. The clinical findings were then compared with those of sporadic HLH patients presenting with hypogammaglobulinemia.

**Results:**

All patients experienced respiratory infections, with two patients showing recurrent episodes. Seizures were observed in 75% of the patients. HLH-related biomarkers were present in all patients. The four patients who did not undergo allogeneic hematopoietic stem cell transplantation (HSCT), all died. Eight variant sites were identified, with 25% located in exon 9 and another 25% in exon 20. The majority (66.67%) of the variants were found in the region responsible for interaction with RAB27α. UNC13D deficiency with hypogammaglobulinemia was associated with a higher frequency of respiratory manifestations, neurological involvement, and an increased mortality rate.

**Conclusions:**

Our study presents the first comprehensive description of the clinical features of UNC13D deficiency with hypogammaglobulinemia. Patients with this condition tend to exhibit more severe clinical manifestations and a poorer prognosis. Allogeneic HSCT may help mitigate immune dysregulation.

## 1 Introduction

Familial hemophagocytic lymphohistiocytosis (FHL) is a hyperinflammatory syndrome caused by pathogenic variants in genes involved in granule-mediated cytotoxicity, including FHL1-5 (1). FHL typically presents in infancy with a high mortality rate, and the only definitive treatment is allogeneic hematopoietic stem cell transplantation (HSCT) (2). Among the various types of FHL, FHL3, caused by variants in the *UNC13D* gene, is the most prevalent form in Asia (3–5). The *UNC13D* gene encodes Munc13-4, a protein essential for the priming and docking of cytotoxic granules in T and NK cells (6, 7). While much research has focused on the immune dysfunction in these cells, the role of humoral immunity in FHL3 remains poorly understood, with limited reports of antibody deficiency in FHL3 and sporadic HLH patients. Given the rarity of hypogammaglobulinemia in FHL3, further investigation is needed to determine whether this deficiency is a common feature or incidental manifestation (8–10).

Our study aims to describe the clinical features, laboratory findings, and immunological results of FHL3 patients with hypogammaglobulinemia. We also reviewed cases of FHL3 with hypogammaglobulinemia from the literature and compared these findings with data on sporadic HLH to better understand the relationship between FHL3 and antibody deficiency.

## 2 Patients and methods

### 2.1 Patients

This study was approved by the Ethics Committee of Chongqing Medical University (No. 2021.138), and written informed consent was obtained from all participants. We retrospectively reviewed 14 patients diagnosed with UNC13D deficiency at our center between January 2012 and June 2024. Of these, two patients (14.29%) met criteria for hypogammaglobulinemia, defined as immunoglobulin G (IgG) levels below the age- and gender-specific reference ranges established at our institution. We collected demographic, clinical, laboratory, and genetic data for these cases. Follow-up data were obtained through medical record review and telephone interviews.

### 2.2 Literature review

A systematic search of PubMed and Web of Science databases was conducted for articles published up to October 13, 2024, using the terms: (“familial hemophagocytic lymphohistiocytosis” OR “hemophagocytic lymphohistiocytosis” OR “HLH” OR “FHL” OR “macrophage activation syndrome” OR “hemophagocytic syndrome”) AND (“hypogammaglobulinemia” OR “antibody deficiency” OR “low IgG” OR “humoral immune deficiency”). Duplicates, animal studies, conference abstracts, and reviews were excluded. This review identified three additional UNC13D-deficient patients with hypogammaglobulinemia from the literature. Furthermore, data on patients with sporadic HLH and hypogammaglobulinemia were extracted. Sporadic HLH was defined as HLH without pathogenic gene mutations, triggered by infections. Ultimately, nine patients with sporadic HLH and hypogammaglobulinemia were identified from six publications (**Supplementary Table S1**).

### 2.3 Statistical analysis

Statistical analysis was conducted by R software (version 4.4.1). The measurement data were expressed in the form of numbers and percentages. For count data, normality was first assessed using the Shapiro-Wilk test (two-sided test, α = 0.10). Data that conformed to a normal distribution were expressed as mean ± standard deviation, while data that did not follow a normal distribution were reported as median and interquartile range. To evaluate the significance of differences, we employed the unpaired t-test and Fisher’s exact test. A p-value of less than 0.05 was defined to be statistically significant.

## 3 Results

### 3.1 Case presentation

Patient 1 (P1), initially described in a previous report (11), presented with recurrent fever and hepatosplenomegaly since 9 months of age. Between ages 1 and 9 years, she required annual hospitalizations for recurrent cough and fever episodes, attributed to suspected respiratory infections. At age 9, she was admitted to our center. Laboratory investigations revealed trilineage cytopenia, hypertriglyceridemia (3.37 mmol/L), hypofibrinogenemia (0.53 g/L), hyperferritinemia (1500 µg/L), and elevated soluble CD25 (>44,000 pg/mL), establishing an HLH diagnosis. A computed tomography (CT) scan demonstrated right lung inflammation. Hypogammaglobulinemia was observed, with IgG at 4.79 g/L (>2 SD below age- and gender-specific means), alongside markedly reduced T-, B-, and NK-cell counts despite normal B-cell proportions. No prior immunosuppressive agents affecting humoral immunity had been administered. Subsequently, without immunoglobulin replacement, her IgG levels progressively declined to a nadir of 2.52 g/L. Genetic testing identified compound heterozygous *UNC13D* variants, confirming FHL3. Chemotherapy per HLH-2004 protocol achieved symptom resolution. At age 13, fever recurred with findings indicative of HLH reactivation. During this episode, she developed convulsive seizures, suggesting CNS involvement. Chemotherapy was reinitiated, followed by unrelated-donor HSCT at age 14. Post-HSCT, symptoms resolved completely with normalization of immunoglobulin levels.

Patient 2 (P2) was admitted to our center at age 3 years with recurrent fever, hepatosplenomegaly, cytopenia, hypofibrinogenemia (0.99 g/L), hyperferritinemia (1965 µg/L), elevated soluble CD25 (38,971 pg/mL), and hemophagocytosis on bone marrow examination, leading to an HLH diagnosis. Genetic analysis confirmed UNC13D deficiency through compound heterozygous *UNC13D* variants. During the HLH episode, he developed hypogammaglobulinemia (IgG 4.98 g/L; >2 SD below age- and gender-specific means) with significantly reduced absolute B-cell counts and proportions. No prior immunosuppressive agents or steroids had been administered. The patient contracted Streptococcus pneumoniae pneumonia despite no history of recurrent respiratory infections. Immunoglobulin levels transiently decreased but normalized spontaneously after symptom resolution without intravenous immunoglobulin (IVIG) therapy. IVIG was withheld because his IgG levels were historically normal, the deficiency was transient, and values were not critically low. He subsequently developed convulsive seizures and right-sided limb weakness suggestive of hemiplegia. HLH-directed chemotherapy per HLH-94 protocol (12) (dexamethasone, etoposide, cyclosporine A) achieved initial remission but failed to prevent recurrence. The patient ultimately died of recurrent HLH at age 8 years.

Patient 3 (P3), as reported by Dr. Rohr (8), was diagnosed with hypogammaglobulinemia (IgG < 5 g/L) at the age of 32, with persistently low IgG levels thereafter. Before experiencing the first episode of HLH at 34 years of age, he suffered recurrent respiratory infections. Genetic analysis confirmed a UNC13D deficiency. Lymphocyte analysis revealed a reduction in marginal zone-like B cells and class-switched memory B cells, indicating functional impairments in the marginal zone and germinal center. Unfortunately, the patient succumbed to HLH.

Patient 4 (P4), as described by Dr. Yazdani (9), presented with fever, anemia, and hepatosplenomegaly at one month of age, along with recurrent diarrhea. At 6 months, the patient developed pneumonia, and a CT scan showed solid lung lesions. Laboratory findings revealed hypogammaglobulinemia (IgG 4.5 g/L) and a reduced proportion of B cells. Despite regular IVIG supplementation, the patient continued to experience recurrent fevers, with progressive worsening of the lung lesions. He was diagnosed with FHL3 at 8 months but tragically succumbed to respiratory failure and bradycardia at 13 months.

Patient 5 (P5), as reported by Dr. Giardino (10), presented with fever and rapidly progressive respiratory failure at the age of 6 years. A CT scan revealed extensive lesions in both lungs. Further evaluations confirmed a diagnosis of HLH accompanied by hypogammaglobulinemia, with the lowest recorded IgG level being approximately 3.15 g/L. Notably, the patient had been healthy prior to this episode, with no history of recurrent infections. Tragically, the patient succumbed to cardiopulmonary failure.

### 3.2 Clinical features of the patients

We summarized the clinical characteristics of these five patients in **Table 1**. All patients experienced respiratory infections (5/5). Neurological involvement was observed in 75% (3/4) of patients in varying forms. Two patients (P1 and P3) had a history of recurrent respiratory infections prior to the diagnosis of FHL3, accounting for 40% of the cohort. However, due to incomplete patient recall, it was difficult to determine whether these infections affected the upper or lower respiratory tract. Epstein-Barr virus (EBV) infection was noted in 40% (2/5) of the patients. Immunoglobulin supplementation was administered to 75% of the patients, although only one received regular therapy. HSCT was performed in only one patient. The mortality rate for patients with UNC13D deficiency with hypogammaglobulinemia reached 80%, rising to 100% among those who did not undergo HSCT.

**Table 1:** Clinical characteristics in UNC13D deficiency with hypogammaglobulinemia.

Clinical characteristics	P1	P2	P3	P4	P5
Gender	F	M	M	M	M
Age at onset of symptoms (months)	9	36	408	1	72
Ethnicity	Chinese	Chinese	Caucasian	Iranian	NA
Fever	Y	Y	NA	Y	Y
Hepatomegaly	Y	Y	NA	Y	NA
Splenomegaly	Y	Y	Y	Y	Y
Respiratory infection	Y	Y	Y	Y	Y
Recurrent respiratory infection	Y	N	Y	N	N
Neurological manifestations	Y (seizures)	Y (seizures, hemiplegia)	NA	Y (seizures)	N
EBV infection	Y	N	Y	N	N
Other pathogens	N	Streptococcus, pneumoniae candida albicans	NA	N	klebsiella pneumoniae, candida albicans
WBC (*10^9/L)	0.62	1.42	NA	4.13	0.8
Neutrophil count (*10^9/L)	0.04	0.29	NA	1.72	0.43
Lymphocyte count (*10^9/L)	0.55	1.07	NA	1.70	0.32
PLT (*10^9/L)	1	15	NA	34	27
Hb (g/L)	44	72	NA	87	78
Ferritin (ug/L)	1500	1965	NA	6171	2550
Triglyceride (mmol/L)	3.37	2.31	NA	2.84	17.56
Fibrinogen (g/L)	0.53	0.99	NA	1.29	0.89
sCD25 (pg/ml)	>44000	38971	NA	NA	NA
NK-cell cytotoxicity	ND	Normal	NA	NA	NA
Hemophagocytosis in bone marrow, spleen, lymph nodes, or liver	N	Y	NA	NA	N
Pulmonary Imaging	Inflammation of the middle lobe of the right lung; small bilateral pleural effusions	Interstitial changes in both lungs	NA	Solid lesion in the right upper lobe of the lung	Extensive solid changes in both lungs, pneumothorax
Chemotherapy	HLH2004	HLH94	NA	N	N
IVIG	Y	N	NA	Y	Y
Other treatment	Antibiotics, Intrathecal chemotherapy, blood transfusion	Antibiotics, Intrathecal chemotherapy, blood transfusion, rehabilitation training	NA	antibiotics	Antibiotics, antifungal, ECMO due to massive pneumothorax
HSCT	Y	N	N	N	N
Prognosis	alive	dead	dead	dead	dead

F, female; M, male; Y, yes; N, no; NA, not accessed; ECMO, extracorporeal membrane oxygenation.

**Figure 1:**
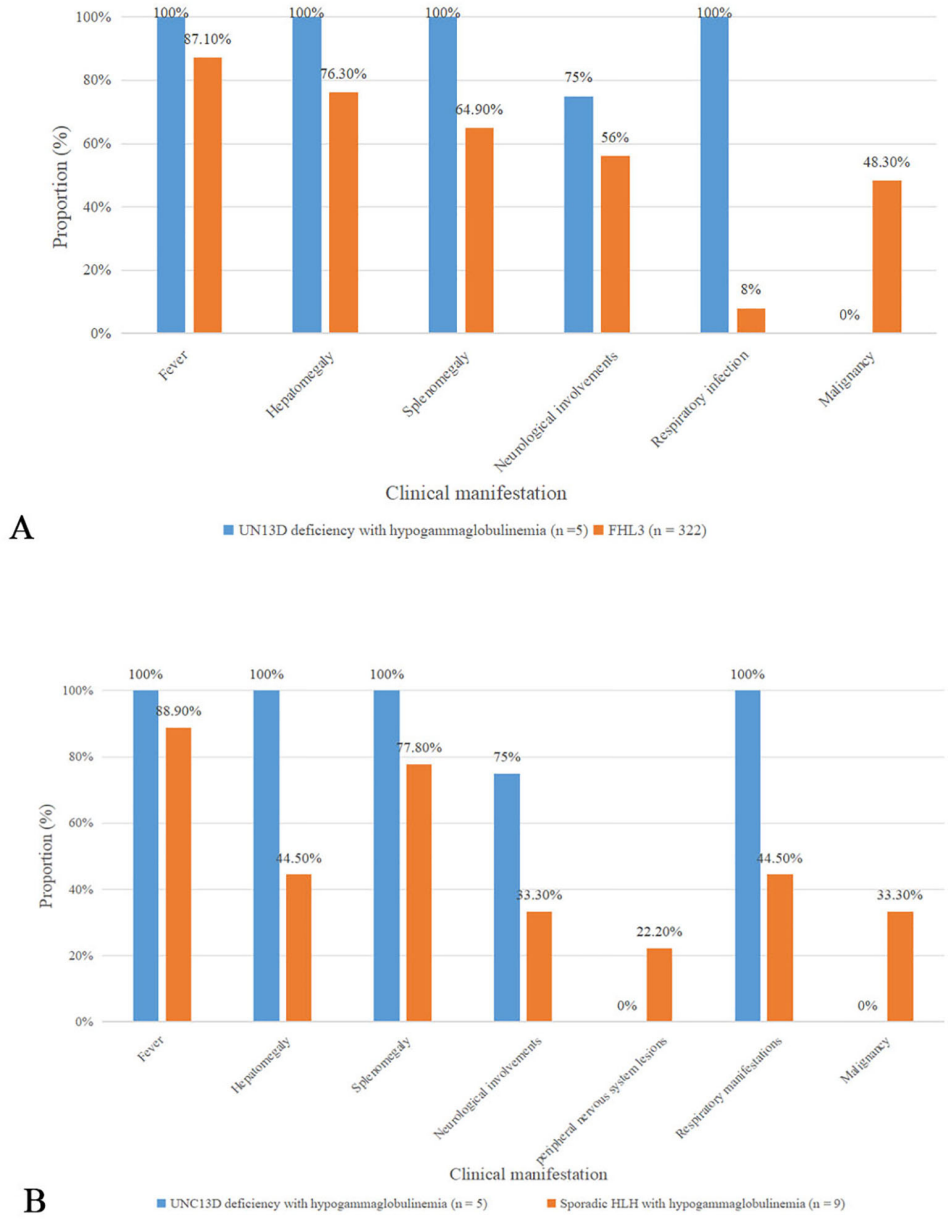
Comparison of clinical characteristics **(A)** Comparison about clinical features bewteen UNC13D deficiency with hypogammaglobulinemia and FHL3 in a systematic review **(B)** Comparison bewteen UNC13D deficiency with hypogammaglobulinemia and sporadic HLH with hypogammaglobulinemia.

Compared to FHL3 data from a systematic review (13), patients with UNC13D deficiency with hypogammaglobulinemia had a higher incidence of neurological involvement (75% *vs*. 56%), a significantly greater frequency of respiratory infections (100% *vs*. 8%; p <0.01), and higher mortality rates (80% *vs*. 48.94%) (**Figure 1A**). In terms of HLH-related biomarkers, these patients exhibited lower median levels of white blood cells, hemoglobin, platelets, and fibrinogen (**Table 2**).

**Table 2:** Comparison about clinical features between UNC13D deficiency with hypogammaglobulinemia and FHL3 in a systematic review (13).

Clinical features	UN13D deficiency with hypogammaglobulinemia (n =5)	FHL3 (n = 322)
Male(%)	4/5 (80%)	110/185 (59.46%)
Fever (%)	4/4 (100%)	155/178 (87.1%)
Hepatomegaly(%)	3/3 (100%)	87/114 (76.3%)
Splenomegaly(%)	5/5 (100%)	74/114 (64.9%)
Neurological involvements(%)	3/4 (75%)	70/125 (56%)
Respiratory infection(%)	5/5 (100%)	4/50 (8%)
Malignancy(%)	0/5 (0%)	44/91 (48.3%)
Cytopenia(%)	4/4 (100%)	23/54 (42.6%)
Mortality(%)	4/5 (80%)	23/47 (48.94%)
WBC absolute count median (IQR) (10^9cells/L)	1.11 (1.34)	6.8 (4.42)
Hb median (IQR) (g/L)	75 (15.25)	84 (29.5)
PLT count median (IQR) (10^9cells/L)	21 (17.25)	34 (51)
Fibrinogen median (IQR) (g/L)	0.94 (0.27)	1.07 (1)
Triglyceride median (IQR) (g/L)	3.11 (4.21)	3.4 (3.85)
Ferritin median(IQR) (ng/mL)	2258 (1606.5)	2353 (4472)

We also summarized data on nine patients with sporadic HLH combined with antibody deficiency reported in the literature (14–19) (**Table 3**, **Supplementary Table S1**). Among these patients, four had EBV infection, three were infected with Blastocystis marneffei, and one suffered from leptospirosis. In three cases, antibody deficiency preceded the diagnosis of HLH, with two of them initially considered for common variable immunodeficiency (CVID). In comparison to sporadic HLH with hypogammaglobulinemia, UNC13D deficient patients with hypogammaglobulinemia presented with an earlier age of onset and were more likely to exhibit hepatosplenomegaly (100% *vs*. 44.5%, and 100% *vs*. 77.8%), respiratory infections (100% *vs*. 44.5%), neurological manifestations (75% *vs*. 33.3%), and a higher mortality rate (80% *vs*. 66.7%) (**Figure 1B**). Peripheral neuropathy was observed in two patients with HLH and antibody deficiency, but was absent in those with UNC13D deficiency with hypogammaglobulinemia. While EBV infection occurred in a similar proportion of both groups (40% *vs*. 44.5%), the mean IgG level was higher in patients with UNC13D deficiency with hypogammaglobulinemia, despite decreased IgG levels in all cases. Unfortunately, these clinical manifestations mentioned above did not have statistical significance, which may be due to the small quantities of cases.

**Table 3:** Comparison between UNC13D deficiency with hypogammaglobulinemia and sporadic HLH with hypogammaglobulinemia.

Clinical features	UNC13D deficiency with hypogammaglobulinemia (n = 5)	Sporadic HLH with hypogammaglobulinemia (n = 9)
Gender, Male (%)	4/5 (80%)	5/9 (55.6%)
Age at onset (years), median (IQR)	3 (0.75, 6)	17 (3.67, 43)
Fever	4/4 (100%)	8/9 (88.9%)
Hepatomegaly	3/3 (100%)	4/9 (44.5%)
Splenomegaly	5/5 (100%)	7/9 (77.8%)
Neurological involvements	3/4 (75%)	3/9 (33.3%)
Peripheral nervous system lesions	0/5 (0%)	2/9 (22.2%)
Respiratory manifestations	5/5 (100%)	4/9 (44.5%)
Malignancy	0/5 (0%)	3/9 (33.3%)
Death	4/5 (80%)	6/9 (66.7%)
EBV infection	2/5 (40%)	4/9 (44.5%)
Chronic active EBV infection	1/5 (20%)	3/9 (33.3%)
Decrease of B cell absolute value or proportion	4/4 (100%)	4/5 (80%)
Decrease of NK cell absolute value or proportion	3/4 (75%)	5/5 (100%)
IgG (g/L)	4.36 ± 0.83	2.83 ± 1.77

### 3.3 Immunological and genetic characteristics of the patients

All patients exhibited mildly reduced IgG levels. The immunological characteristics are summarized in **Table 4**. Three out of 4 patients (75%) showed a reduced proportion of B cells, while half (2/4) demonstrated a decreased proportion of NK cells.

**Table 4:** Immunological characteristics in UNC13D deficiency with hypogammaglobulinemia.

Immunological features	P1	P2	P3	P4	P5
IgG (g/L)	4.79↓ (5.28~21.9)	4.98↓ (5.28~21.9)	decreased	4.5 ↓(7~16)	3.15↓
IgA (g/L)	<0.0667↓ (0.51~2.59)	0.697 (0.61~3.45)	NA	0.36↓ (0.41~2.97)	0.46
IgM (g/L)	0.304↓ (0.48~2.26)	0.566 (0.48~2.26)	NA	0.35 ↓ (0.4~2.3)	0.58
CD4^+^T cells (%)	52.76% (27%~53%)	36.04% (27%~53%)	NA	57%↑ (35%~56%)	34% (26.17%~40.76%)
CD4^+^T cells (cells/ul)	400.16↓ (621.39~1258.00)	389.23↓ (902.39~2253.93)	NA	NA	109↓ (685.89~1357.82)
CD8^+^T cells (%)	28.01% (19%~34%)	33.08% (19%~34%)	NA	25% ↑ (12%~23%)	13%↓ (19.68%~34.06%)
CD8^+^T cells (cells/ul)	187.65↓ (508.71~1050.13)	357.26 ↓ (580.42~1734.71)	NA	NA	42 ↓ (517.67~1125.24)
B cells (%)	10.12% (10%~31%)	10.47%↓ (13.23%~26.39%)	NA	0.5% ↓ (11%~41%)	8% ↓ (10.21%~20.12%)
B cells (cells/ul)	42.46↓ (247.05~578.16)	113.08↓ (461.19~1455.91)	NA	NA	26↓ (279.78~622.56)
NK cells (%)	3.64% ↓ (4%~26%)	6.76%↓ (7.21%~20.90%)	NA	3% (3%~14%)	22%↑ (10.21%~20.12%)
NK cells (cells/ul)	41.68↓ (202.54~583.53)	73.01↓ (269.94~1053.21)	NA	NA	70↓ (257.53~726.73)

NA, not accessed, the numbers between parentheses are the normal ranges related to the age and gender in different laboratories.

The symbol "↓"  means decreased, and the symbol "↑" means increased.

**Figure 2:**
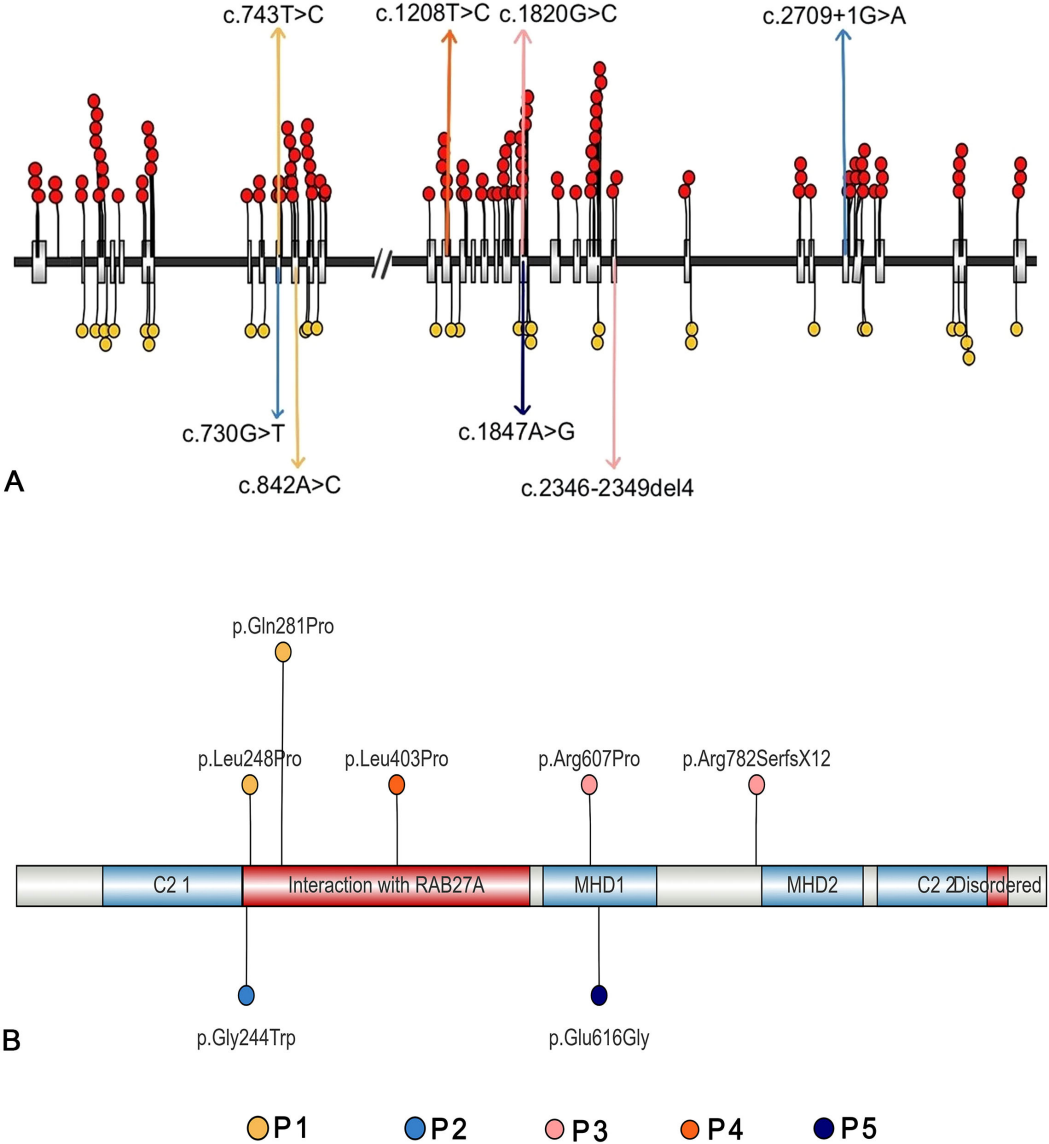
Genetic features of the five patients with UNC13D deficiency with hypogammaglobulinemia **(A)** The mutation sites of the five patients, along with all published pathogenic mutations (red circles) and likely pathogenic mutations (yellow circles) **(B)** The affected acid sites of the five patients.

The details of the variants are summarized in **Table 5**. Three patients carried compound heterozygous variants, while two had homozygous variants. A total of eight variant sites were identified, with 75% classified as missense variants, 12.5% as splice site variants, and 12.5% as frameshift variants. Notably, 25% (2/8) of the variants were located in exon 9, and another 25% (2/8) in exon 20. Regarding protein structure, 66.67% of the patients had variants in the region responsible for interaction with RAB27α, while 33.33% had variants within the MHD1 domain (**Figure 2B**, **Table 6**).

**Table 5:** Genetic characteristics in UNC13D deficiency with hypogammaglobulinemia.

Genetic mutations	P1	P2	P3	P4	P5
Location 1	exon9	intron	exon20	exon14	exon20
Mutation 1	c.743T>C, p.Leu248Pro	c.2709 + 1G>A	c.1820G>C, p.Arg607Pro	c.1208T>C, p.Leu403Pro	c.1847A>G, p.Glu616Gly
Location 2	exon10	exon9	exon24	/	/
Mutation 2	c.842A>C, p.Gln281Pro	c.730G>T, p.Gly244Trp	c.2346-2349del4, p.Arg782SerfsX12	/	/
Mutation type	Missense	Missense+Splice error	Missense+Frameshift	Missense	Missense
Genotype	Compound heterozygous	Compound heterozygous	Compound heterozygous	Homozygous	Homozygous

**Table 6:** Comparison about genetic features bewteen UNC13D deficiency with hypogammaglobulinemia and FHL3 in a systematic review (13).

Mutation features		UN13D deficiency with hypogammaglobulinemia	FHL3
Mutation type (%)	Missense	75	20.47
	Nonsense	0	10.87
	Deletion	0	0.63
	Insertion	0	0.31
	Frameshift	12.5	13.59
	Splice error	12.5	35.0
	Inversion	0	5.94
	Unknown	0	13.28
Genotype (%)	Homozygous	40	36.68
	Heterozygous	0	15.67
	Compound heterozygous	60	47.65
Mutation site (%)	Exon	87.5	53.39
	Intron	12.5	46.61
Domain of UNC13D protein (%)	C2A	0	33.3
	Interaction with RAB27α	66.67	6.8
	MHD1	33.33	13.6
	MHD2	0	19.7
	C2B	0	26.5

Regarding genetic variants, patients who had UNC13D deficiency with hypogammaglobulinemia were more likely to carry missense variants (75% *vs*. 20.47%), have a higher frequency of mutated sites within gene exons (87.5% *vs*. 53.39%), and show a greater likelihood of variants affecting the domain responsible for interaction with RAB27α (**Figure 2**, **Table 6**).

## 4 Discussion

Our study identified a significantly higher incidence of respiratory complications in patients who had UNC13D deficiency with hypogammaglobulinemia compared to those with FHL3 and sporadic HLH with antibody deficiency. This finding may be attributed to the increased susceptibility to respiratory infections in antibody-deficient individuals. Although only two patients had a documented history of recurrent respiratory infections, rapid disease progression or early mortality may have obscured this feature in others. Recurrent bacterial infections are strongly associated with antibody deficiency and can lead to chronic lung conditions such as bronchiectasis and fibrosis (20–24). However, none of these five patients developed such complications. Potential contributing factors may include relatively higher IgG levels compared to classic antibody deficiencies (e.g., X-linked agammaglobulinemia [XLA]), appropriate antimicrobial therapy, limited follow-up duration, or immune reconstitution achieved through HSCT (25).

Neurological complications were common in patients with UNC13D deficiency, with all affected individuals presenting with convulsive seizures, which is consistent with previous reports that FHL3 patients often experience central nervous system involvement, including encephalopathy and seizures (13, 26). In some atypical cases of FHL3, isolated neurological symptoms may manifest without systemic inflammation, possibly due to residual protein function that mitigates systemic activation (27–29). However, in our cohort, all patients initially presented with systemic HLH, with neurological symptoms emerging later. This suggests a more severe dysfunction of Munc13–4 in these cases with hypogammaglobulinemia.

The higher mortality rate observed in patients with UNC13D deficiency with hypogammaglobulinemia, compared to those with FHL3 or HLH associated with antibody deficiency, raises the question of whether hypogammaglobulinemia could serve as a prognostic risk factor in FHL3. This hypothesis was proposed because antibody deficiency, especially without conventional alternative treatment, may lead to increased susceptibility to infection, thereby triggering HLH reactivation, and previous literature has suggested that HLH reactivation is associated with increased mortality (12, 30, 31). Previous studies have highlighted the efficacy of IVIG as a safe immunomodulator for inducing remission in HLH, particularly in secondary HLH cases (32–35). Tan CJ et al. (36) further demonstrated a strong association between IVIG use and reduced mortality in a systematic review. In our cohort, three of the five patients received IVIG, with only one (P4) receiving regular monthly supplementation. However, due to the small sample size, it remains unclear whether regular IVIG treatment provides significant benefits for FHL3 patients, and larger-scale studies are needed to clarify its potential advantages in this population. Simultaneously, antibody deficiency may obscure the clinical diagnosis of FHL, resulting in diagnostic delays, postponed HSCT, and poorer patient outcomes.

*UNC13D* variants in patients with hypogammaglobulinemia were predominantly found in exons 9 and 20, which affect the RAB27α interaction domain, crucial for granule-mediated cytotoxicity. This interaction is essential for the fusion of late and recycling endosomes, enabling proper lytic granule function (37, 38). Elstak et al. (39) demonstrated that while degranulation failed to restore in cells carrying a mutated RAB27α-binding motif in Munc13-4, this function was recovered to near-wild-type levels when the RAB27α-binding motif remained intact. Thus, the Munc13-4-RAB27a interaction is essential for degranulation. Variants that disrupt this complex severely impair cytotoxicity, which correlates with worse clinical outcomes and higher mortality (39). In our cohort, the mortality rate was higher than that of FHL3 patients as evaluated in a systematic review (13). These findings suggest that variants in *UNC13D* affecting RAB27α binding may contribute to poorer clinical outcomes, a hypothesis that warrants further validation.

Previous studies have shown that HLH activity itself can lead to secondary antibody deficiency. Inflammatory cytokines such as IFN-γ and TNF inhibit B-cell maturation and reduce immature B-cell populations, including CD19+ and CD19+CD10+ subsets (40, 41). IFN-γ, through Interferon Regulatory Factor 1(IRF1), contributes to the reduction of class-switched memory B cells, plasma cells, and immunoglobulin levels, impairing all stages of B-cell development. Notably, antibody deficiency can be reversed once T-cell hyperactivation is controlled (40, 42, 43). While the mechanism underlying antibody deficiency in UNC13D deficiency remains unclear, insights can be gleaned from mutations in other vesicle transport-related genes, such as STX11. Kögl et al. (44) observed that STX11-mutant mouse models exhibited significantly reduced CD40L expression on CD4+T cells, along with diminished secretion of IL-2 and IL-10. These alterations compromise T cell-dependent B cell activation, ultimately impairing the B cell response to antigens. Consequently, secondary B cell defects arise, characterized by the absence of germinal centers, defective class-switch recombination, profoundly reduced levels of IgA and IgG, and compromised affinity maturation.Similar mechanisms may be involved in UNC13D deficiency (44–46).

Our study has limitations, including a small sample size, retrospective design, and incomplete immunological assessments in some patients. Immunoglobulin levels were not consistently measured before HLH onset, complicating the determination of whether antibody deficiency is primary or secondary to HLH. Larger cohort studies with comprehensive immunological evaluations are needed to clarify this relationship.

## 5 Conclusion

Hypogammaglobulinemia in UNC13D deficiency, a rare manifestation of FHL3, was associated with higher risks of respiratory infections, neurological complications, and mortality compared to previously described FHL3 cohorts, suggesting a more severe clinical course and poorer prognosis. Notably, variants in the UNC13D RAB27α-interaction domain were enriched among patients with hypogammaglobulinemia. Given limited research on humoral immunity impairment in HLH, we recommend routine immunoglobulin screening in HLH patients. This approach may identify additional FHL3 cases with antibody deficiency and enhance our comprehension of the complex interplay between humoral immunity and HLH.

## Data Availability

The original contributions presented in the study are included in the article/**Supplementary Material**. Further inquiries can be directed to the corresponding authors.
