# Supplementary material for: Clinical and genetic features of UNC13D deficiency with hypogammaglobulinemia
Source: Front Immunol. 2025 Aug 18;16:1628507. doi: 10.3389/fimmu.2025.1628507 (PMC12399691; doi:10.3389/fimmu.2025.1628507)
Supplement: Supplementary file 1 [file DataSheet1.pdf]

Supplemental Table S1. Clinical and immunological characteristics in sporadic HLH with hypogammaglobulinemia

Patient	Age of onset (years) (HLH)	sex	clinical features	aetiology	IgG(g/L) (Normal range)	IgA(g/L) (Normal range)	IgM(g/L) (Normal range)	IgE(IU/ML) (Normal range)	C3(g/L) (Normal range)	C4(g/L) (Normal range)	CD3+4+(cells/ul) (Normal range) or proportion	CD3+8+(cells/ul) (Normal range) or proportion	B cell (cells/ul) (Normal range) or proportion	NK Abs(cells/ul) (Normal range)or proportion	gene	WBC*10^9/L	PLT*10^9/L	HB (g/L)
1[13]	0.25	Male	Fever, cough, weight loss, dyspnea, malnutrition, diarrhea, hepatomegaly, splenomegaly; Complications: HLH, DIC, MODS, Septic [illegible]	Talaromyces marneffei	2.23↓ (3.22-7.18)	0.16 (0.13-0.35)	0.89 (0.23-0.91)	5 (0-15)	0.51↓ (0.74-1.38)	0.14 (0.1-0.35)	ND	ND	ND	ND	ND	NA	NA	NA
2[13]	3.67	Male	Fever, cough, diarrhea, dyspnea, skin lesion, lymphadenopathy, hepatomegaly, splenomegaly; Complications: HLH, DIC, MODS, Septic shock, ARDS, Plastic [illegible]	Talaromyces marneffei	4.43↓ (4.53-9.16)	0.62 (0.2-1.0)	0.28 (0.19-1.46)	5 (0-60)	0.86↓ (0.9-1.8)	0.2 (0.1-0.4)	107.66↓ (345-2350)	178.08↓ (314-2080)	167.45↓ (240-1317)	47.86↓ (210-1514)	ND	NA	NA	NA
3[13]	1.83	Male	Fever, cough, diarrhea, skin lesion, lymphadenopathy, hepatomegaly, splenomegaly; Complications: HLH, Septic shock;	Talaromyces marneffei	<0.33↓ (3.82-10.58)	<0.07↓ (0.14-1.14)	0.57 (0.4-1.28)	<5 (0-60)	1.29 (0.8-1.5)	0.15 (0.12-0.4)	744.29 (345-2350)	214.15↓ (314-2080)	1112.15 (240-1317)	79↓ (210-1514)	ND	NA	NA	NA
4[14]	17	Female	Decreased IgG2 level (13y); decreased IgA level (14y); hepatosplenomegaly, chronic active EBV infection, chronic active hepatitis (15y), HLH (17y), chronic inflammatory demyelinating polyneuropathy;	EBV	3.38↓	0.46↓	1.25↓	NA	NA	NA	1103（700-1100）↑	1008（500-900）↑	32↓(200-400)	48↓（200-400）	ND	NA	↓	↓
5[15]	43	Male	CVID, increased liver enzyme levels, cholelithiasis, a cachectic appearance, Hodgkin's lymphoma (42y) ,HLH(43y), myopathy(appeared due to dexamethasone);	EBV	around 5↓	NA	NA	NA	NA	NA	NA	NA	NA	NA	ND	1.7↓	9↓	37↓
6[16]	17	Male	fatigue, myalgias, fever, and pancytopenia, hepatitis, splenomegaly, encephalitis, peripheral neuropathy, facial palsy diplopia, multiple cerebellar and cerebral lesions and demyelinating lesions in the thoracic spine (MRI) , lymphadenopathy, chronic active Epstein-Barr virus disease;	EBV	2.25↓-21.8	NA	NA	NA	NA	NA	79%↑	13%↓	0.5%↓	1.5%↓	ND	↓	↓	↓
7[16]	5	Female	fever, sinusitis, and lymphadenopathy, EBV-positive polymorphic B cell lymphoma, chronic active Epstein-Barr virus disease;	EBV；an atypical mycobacteria infection (after HSCT)	4.32↓	NA	NA	NA	NA	NA	ND	ND	ND	ND	ND	NA	NA	NA
8[17]	61	Female	malaise, abdominal pain, diarrhea, fever, splenomegaly, edema, azotemia,oliguria, swollen kidneys, generalized seizure, comatose, type II diabetes mellitus, [illegible]	Leptospirosis	0.0124↓	<0.071↓	<0.072↓	32.7	Normal	Normal	NA	NA	NA	NA	ND	0.0013↓	0.041↓	101↓
9[18]	68	Female	Thymoma, CVID, anaemia, weight loss, fatigue, hypogammaglobulinemia, HLH;	bacteria,fungi, CMV	3.55↓	0.05↓	0.02↓	≤0.05	NA	NA	24.7%↓	40.3%↑	CD19 0.3% CD20 4.6%↓	17.9%↓	ND	9.7	164	86

Patient	ferritin(ug/L)	sCD25 (U/ML)	Triglycerides (g/L)	fibrinogen (g/L)	activity of NK cell	AST (U/L)	ALT (U/L)	fever	Hepatomegaly	Splenomegaly	Neurological manifestations	Respiratory manifestations	Malignancy	pathology	treatment	HSCT	prognosis	the order of hypogammaglobulinemia and HLH
1[13]	NA	NA	NA	NA	NA	NA	NA	Y	Y	Y	N	Y	N	NA	No antifungal treatment	N	Dead	No obvious order
2[13]	NA	NA	NA	NA	NA	NA	NA	Y	Y	Y	N	Y	N	NA	Fluconazole, Voriconazole, Itraconazole	N	Dead	No obvious order
3[13]	NA	NA	NA	NA	NA	NA	NA	Y	Y	Y	N	Y	N	NA	Caspofungin	N	Dead	No obvious order
4[14]	NA	NA	NA	NA	Normal	NA	NA	Y	Y	Y	Y（intermittent episodes of a steroid responsive demyelinating neuropathy, chronic inflammatory demyelinating polyneuropathy）	N	N	The cells of the lymphoid infiltrate in the liver and spleen show strong positivity for EBV encoded RNAs (EBERs) ; Erythrophagocytosis in the spleen and [illegible]	steroids, IVIG, aciclovir，splenectomy,α-interferon	N	Alive (persistent EBV infection and recurrent haemolysis)	hypogammaglobulinemia before the episode of HLH
5[15]	>50000↑	NA	↑	NA	NA	104↑	90↑	N	N	Y	N	N	Y	Y（lymphohistiocytic aggregates with hemophagocytosis in the bone marrow)	ABVD (Adriamycin, bleomycin, vincristine and dexamethasone) chemotherapy, ICE chemotherapy, monthly IVIG, HLH2004, HSCT	Y	Dead	hypogammaglobulinemia before the episode of HLH
6[16]	NA	NA	NA	NA	NA	NA	NA	Y	N	Y	Y(encephalitis, peripheral neuropathy, facial palsy diplopia, multiple cerebellar and cerebral lesions and demyelinating lesions in the thoracic spine)	N	N	hemophagocytosis in the lymph node and spleen; ; biopsies of a lymph node and liver were positive for EBV RNA	acyclovir, intravenous immune globulin (IVIG), and corticosteroids, IFN-β, cyclosporine, splenectomy, azathioprine etoposide, cytosine arabinoside, cyclophosphamide, antithymocyte globulin, and total body irradiation ,HSCT	Y	Alive	No obvious order
7[16]	NA	NA	NA	NA	NA	NA	NA	Y	N	N	N	Y	Y	hemophagocytosis in the bown marrow, A lymph node biopsy showed EBV-positive B cell lymphoproliferative disease	etoposide, dexamethasone, and cyclosporine, rituximab, autologous EBV-specific cytotoxic T cells treatment, chemotherapy (vincristine, doxorubicin, methotrexate and prednisone), HSCT	Y	Alive	No obvious order
8[17]	9152↑	NA	4.14↑	NA	NA	190↑	78↑	Y	N	Y	Y（generalized seizure, comatose）	N	N	hemophagocytosis in the bone marrow	hemodialysis, ceftriaxone, metronidazole, doxycyclin	N	Dead	No obvious order
9[18]	512	3995	1.58	1.92	NA	107	58	Y	N	N	N	N	Y	hemophagocytosis in the bone marrow	Tumor resection, Ganciclovir, Itraconazole, vancomycin, cefozopran,sulfamethoxazole, trimethoprim, HLH2004, G-CSF	N	Dead	hypogammaglobulinemia before the episode of HLH

Y, yes; N, no; NA, not accessed; ND, not detected;
